# Stent-alone treatment of unruptured vertebral artery fusiform aneurysms: A comparison of flow diverter and conventional stents

**DOI:** 10.3389/fneur.2022.1012382

**Published:** 2022-11-02

**Authors:** Wenqiang Li, Wei Zhu, Yanmin Wang, Yapeng Zhao, Yang Wang, Xianzhi Liu, Yisen Zhang

**Affiliations:** ^1^Department of Neurosurgery, The First Affiliated Hospital of Zhengzhou University, Zhengzhou University, Zhengzhou, China; ^2^Department of Interventional Neuroradiology, Beijing Neurosurgical Institute and Beijing Tiantan Hospital, Capital Medical University, Beijing, China; ^3^Department of Neurosurgery, Beijing Chaoyang Hospital, Capital Medical University, Beijing, China

**Keywords:** vertebral artery fusiform aneurysms, stent alone treatment, flow diverters, conventional stents, comparison

## Abstract

**Background:**

Treatment of vertebral artery fusiform aneurysms (VAFAs) is complex and controversial. This study aimed to compare the safety and efficacy between flow diverter and conventional stents in patients with VAFAs undergoing endovascular stent-alone treatment (SAT).

**Methods:**

Thirty-six patients with 36 VAFAs who underwent SAT between January 2014 and December 2018 were retrospectively analyzed. Patient and aneurysm characteristics, procedural details, complications, and angiographic and clinical outcomes were compared between flow diverter stent patients (*n* = 22) and conventional stent patients (*n* = 14).

**Results:**

More branches covered with stent were found in the conventional stent group (88.9 vs. 33.3%; *p* = 0.008). The number of stents placed was significantly higher in the conventional stent group (1.57 ± 0.76 vs. 1.09 ± 0.29; *p* = 0.016). The proportion of patients with significant or moderate stasis within the aneurysm immediately after stent placement was higher in the flow diverter stent group (95.5 vs. 57.1%; *p* = 0.004). The proportion of patients with complete obliteration or only a residual neck on follow-up angiography was significantly higher in the flow diverter stent group (86.3 vs 50.0%; *p* = 0.047). However, the incidence of parent artery stenosis or occlusion was also higher in the flow diverter stent group (27.3% vs. zero; *p* = 0.032). The rate of complications did not significantly differ between the groups.

**Conclusions:**

SAT was safe and effective in patients with VAFAs. Flow diverter stents are associated with a significantly better complete occlusion rate than conventional stents; however, they are also associated with an increased risk of parent artery stenosis.

## Introduction

Fusiform intracranial aneurysms are defined as circumferential dilatations of a cerebral artery without an ostium or neck (1, 2). They are most frequently located in the vertebral artery. The annual incidence of vertebral artery fusiform aneurysms (VAFAs) is low (with international literature reporting a rate of only 1/100,000 to 1.5/100,000), but is recognized as an important cause of stroke (3, 4). VAFAs can be associated with subarachnoid hemorrhage and nerve compression, as well as intracranial infarcts due to the progression of the aneurysm or clot migration. Considering the risk of rupture and progression, it is necessary to take more aggressive treatment for the unruptured VAFAs. Endovascular treatment is an alternative to open neurosurgical treatment and constitutes a definitive modality for the treatment of intracranial aneurysms. However, endovascular treatment of VAFAs is still challenging and controversial. Current endovascular therapies for VAFAs mainly include parent artery occlusion and reconstructive techniques. If vessel trapping was not feasible, stent-assisted reconstructive techniques, including stent-alone treatment (SAT) and stent-assisted coiling techniques, were considered. SAT was available only in special cases, such as the aneurysm without adequate dilation for coil placement, the aneurysm with important branches, or the blood flow was well established by the placement of a single or multiple stent(s) alone without the need for further coiling. The relevant literature concerning the VAFAs treated with SAT are case reports or small case series (5–7). With the improvements in stents, flow diverter stent is efficient, while they are associated with the risk of ischemia, especially when vital arterial branches are covered (8). Currently, studies directly contrasting SAT of different stents (flow diverter and conventional stents) in patients with VAFAs are rare. Therefore, we performed the study to compare the safety and efficacy between flow diverters and conventional stents in VAFA patients undergoing endovascular SAT.

## Materials and methods

This retrospective study was approved by the institutional review board of Beijing Tiantan Hospital. All patients or their family members provided verbal informed consent.

### Patient selection

Patients diagnosed with VAFA who underwent SAT from January 2014 to December 2018 and follow-up angiography in our institution were eligible for inclusion. The detailed inclusion and exclusion criteria are listed in the Supplementary Table 1. Patient age, sex, body mass index, clinical presentation, smoking, drinking, hypertension, diabetes mellitus, hyperlipidemia, modified Rankin Scale (mRS) score, aneurysm size, and arterial branch anatomy were recorded. Treatment details, complications, and angiographic and clinical outcomes were also recorded.

### Endovascular procedures

Dual antiplatelet therapy (aspirin 100 mg and clopidogrel 75 mg) was administered daily for at least 3 days before treatment in patients who underwent conventional stenting and at least 5 days before treatment in patients who underwent flow diversion stenting. Conventional stents used in this study included the Enterprise stent (Cordis Neurovascular, Miami, FL, USA) and LVIS stent (MicroVention, Inc., Tustin, CA, USA). The flow diverter stent used was the Pipeline embolization device (Medtronic, Dublin, Ireland). All procedures were performed under general anesthesia and full heparinization (target-activated clotting time, 250–300 s). For conventional stenting procedures, a triaxial system was used to place a guiding catheter into the vertebral artery and deploy stents across the aneurysmal neck. Flow diverter stents were introduced through a Marksman microcatheter (Medtronic), delivered to the vertebral artery, and then deployed. The number of stents deployed depended on aneurysm location, vertebral artery tortuousness, and angiographic findings after deployment of the first stent. If a single stent was not sufficient to remodel the blood flow adequately, a multiple stent technique was performed. A microcatheter was re-advanced to the initial position over the stent loading wire, and a second stent was advanced into the vertebral artery, centered within the stent, across the neck of the aneurysm, reinforcing the expected flow-diversion effect. If the two stents could not cover the aneurysm or the reduced blood flow is not sufficient, then a third stent was guided to the distal end of the second stent, deployed within the previously placed stents, again centering over the neck of the aneurysm. Several endovascular techniques (use of wires, catheters, or balloon angioplasty) were used if the device did not expand adequately. Immediately after stent placement, angiography was performed to evaluate blood flow, which was classified as significant stasis, moderate stasis, or no stasis. Patients who underwent conventional stenting remained on dual antiplatelet therapy for 6 weeks and then aspirin alone for an additional 6 months. Those who underwent placement of a flow diverter stent remained on dual antiplatelet therapy for 3 months and then aspirin alone for an additional 12 months.

### Clinical and angiographic follow-up

Imaging follow-up was performed using digital subtraction angiography approximately 6 months after stenting. Subsequent follow-up was performed annually using magnetic resonance angiography or computed tomography angiography. The occlusion rate was evaluated using the O'KellyMarotta (OKM) grading scale (9). Recurrence was defined as an aneurysm that showed an increased percentage of contrast filling within the aneurysmal sac on follow-up angiography. All imaging studies were evaluated independently by two neurointerventionalists with more than 5 years of experience. Any disagreements were resolved by a third neurointerventionalist with 10 years of experience. Clinical outcomes were evaluated by determining the mRS score at follow-up visits or *via* telephone interviews.

### Statistical analysis

Statistical analyses were performed using SPSS software version 24.0 (IBM Corp., Armonk, NY, USA). Continuous data are presented as means with standard deviation and were compared using the Wilcoxon rank sum test. Categorical data are presented as numbers with percentages and were compared using the χ2 test or Fisher exact test. *P* < 0.05 was considered significant.

## Results

### Patient and aneurysm characteristics

Thirty-six patients, each with a single VAFA, were included for analysis. Twenty-two (61.1%) were treated using flow diverter stent and 14 (38.9%) using a conventional stent. Patient and aneurysm characteristics of the two groups are shown in Table 1. Although a higher proportion of patients in the flow diverter stent group were women and the proportion of asymptomatic patients was higher in the conventional stent group, the differences were not significant.

**Table 1:** Patient and aneurysm characteristics.

	**Flow diverter stent (*n =* 22)**	**Conventional stent (*n =* 14)**	***P* value**
Age	51.27 ± 8.05	48.21 ± 7.85	0.071
Female, %	6 (27.3)	2 (14.3)	0.361
Body mass index	25.61 ± 2.35	25.65 ± 4.01	0.673
Presentation			0.208
Asymptomatic, %	5 (22.7)	18 (50.0)	
Headache, %	4 (59.1)	44 (28.6)	
Mass effect, %	2 (4.5)	0 (0.0)	
Weakness of limbs, %	1 (13.6)	3 (21.4)	
Risk factors			
Smoking, %	7 (31.8)	5 (35.7)	0.809
Drinking, %	7 (31.8)	4 (28.6)	0.837
HTN, %	8 (36.4)	7 (50.0)	0.418
DM, %	1 (4.5)	0 (0.0)	0.418
HLD, %	1 (4.5)	1 (7.1)	0.740
Branch coming from aneurysm	15 (68.2)	9 (64.3)	0.809
Aneurysm size	10.73 ± 4.18	9.91 ± 4.19	0.519
Pre-procedure mRS	0.77 ± 0.43	0.71 ± 0.61	0.642

HTN, hypertension; DM, diabetes mellitus; HLD, hyperlipidemia; mRS, modified Rankin scale.

### Treatment details, complications, and angiographic outcomes

**Figure 1:**
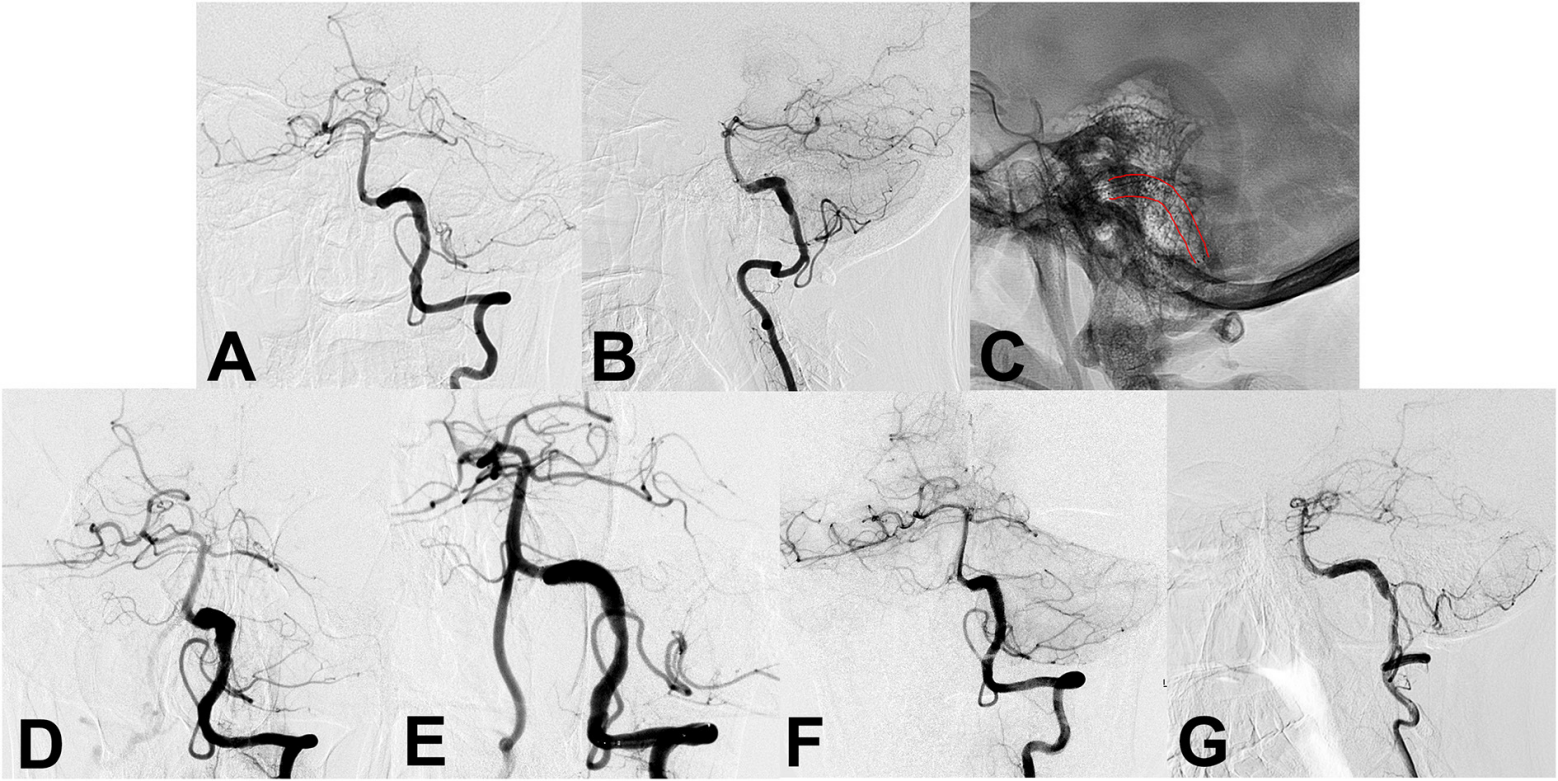
**(A,B)** Preoperative angiography shows an unruptured vertebral artery fusiform aneurysm. **(C)** Intraoperative angiography shows the successful placement of a single conventional stent (lines). **(D,E)** Angiography immediately after stent placement showed residual flow inside the aneurysm. **(F,G)** Follow-up angiography 6 months after the procedure continued to show residual aneurysm.

**Figure 2:**
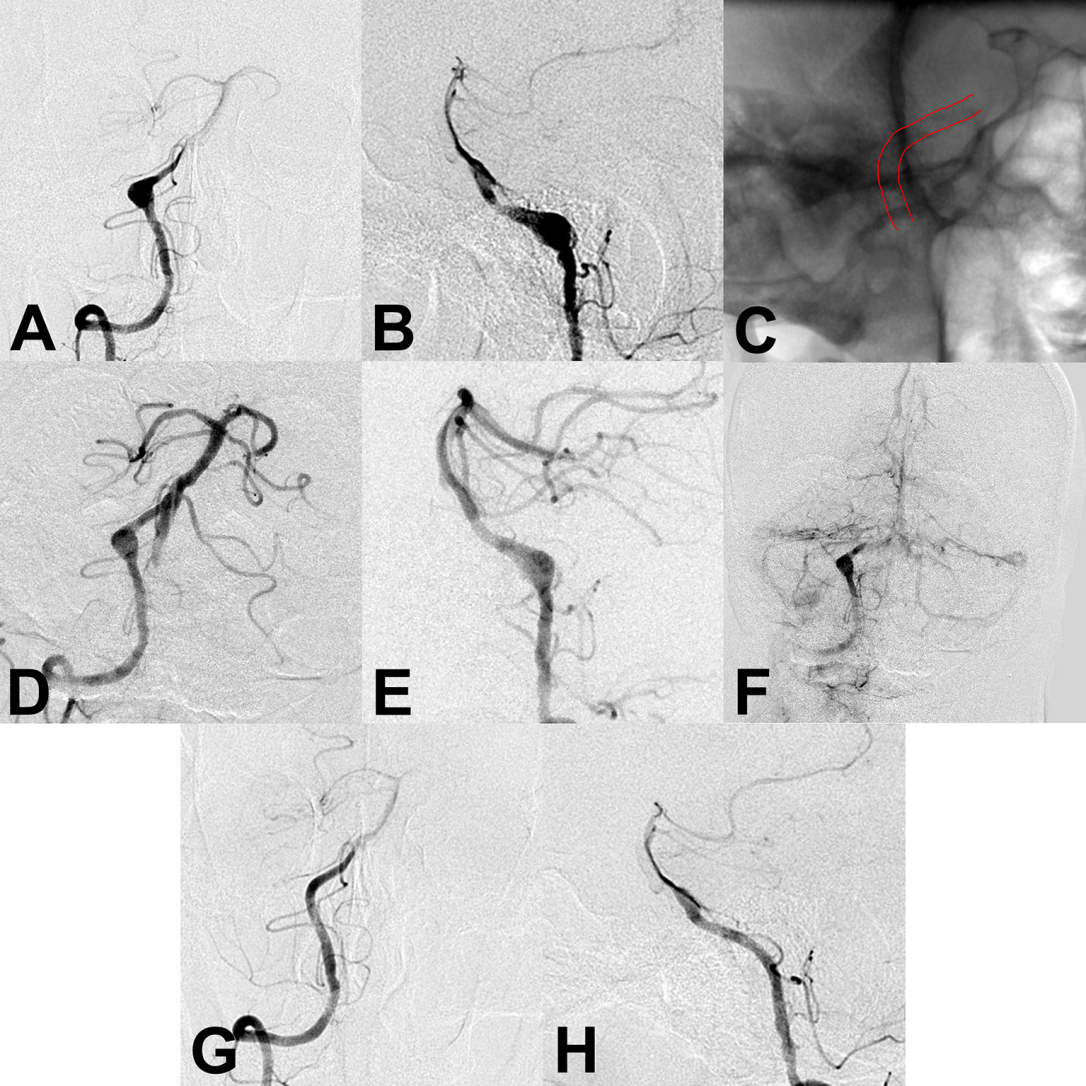
**(A,B)** Preoperative angiography showed an unruptured vertebral artery fusiform aneurysm. **(C)** Intraoperative angiography showed successful deployment of a flow diverter stent (lines). **(D–F)** Angiography immediately after the procedure showed a residual aneurysm with significant stasis. **(G,H)** Follow-up angiography 6 months after the procedure showed complete aneurysm obliteration.

**Figure 3:**
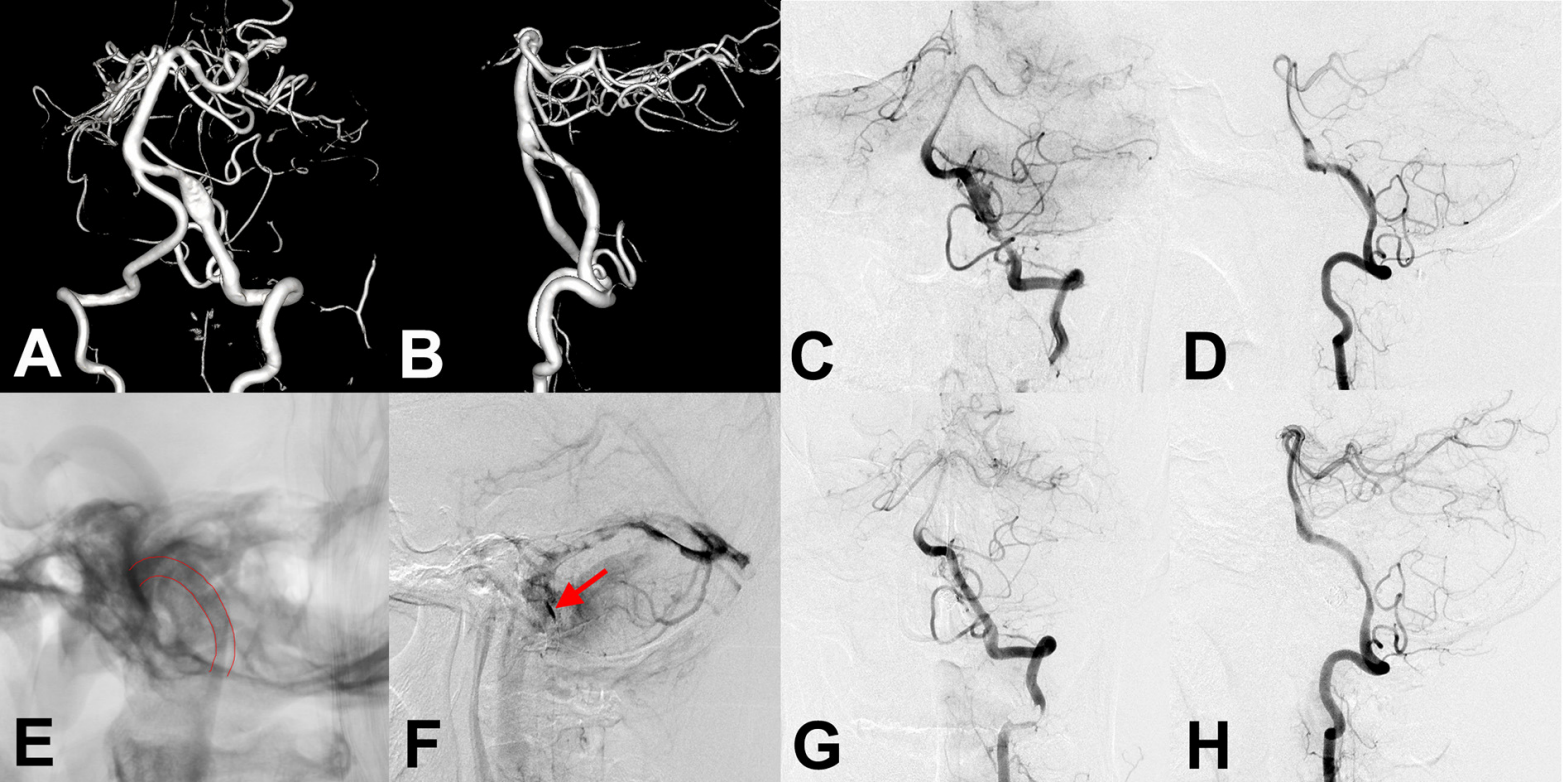
**(A,B)** Preoperative anteroposterior and lateral angiography showed an unruptured vertebral artery fusiform aneurysm. **(C,D,F)** Immediate angiography after stenting showed residual aneurysm with significant stasis. **(E)** Flow diverter stent showed successful deployment in intraoperative angiography (lines). **(G,H)** Follow-up angiography at 6 months after the procedure showed an aneurysm occlusion completely, while accompanied by parent artery stenosis. The patient showed no clinical symptoms.

**Figure 4:**
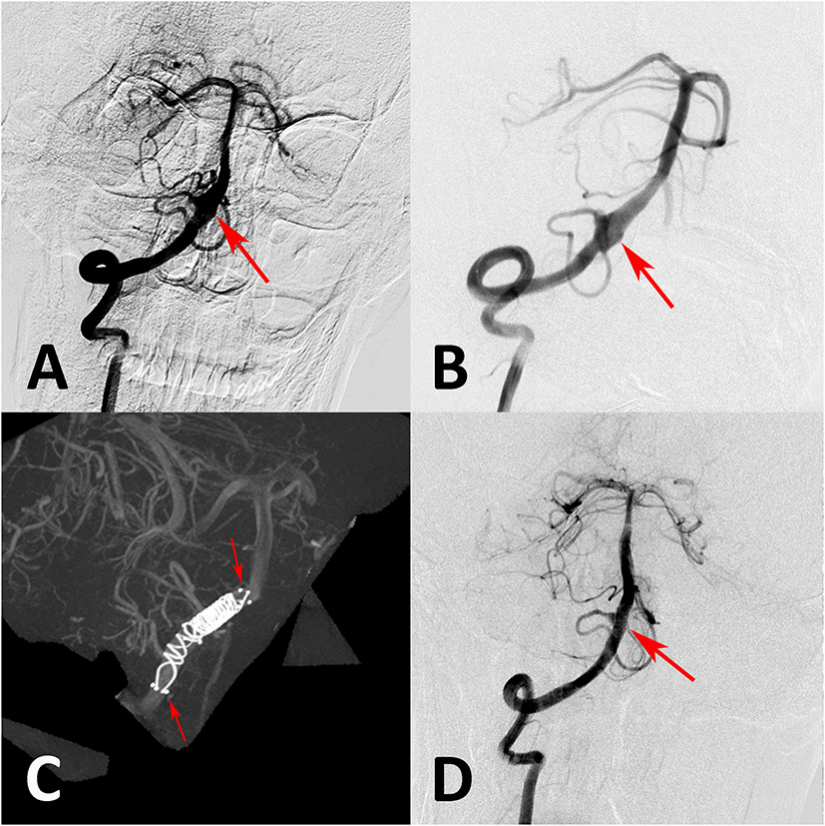
**(A)** Preoperative angiography showed an unruptured vertebral artery fusiform aneurysm. **(B)** Immediate angiography after stenting showed aneurysm persisted. **(C)** Overlapping stent technique with double conventional stents showed in intraoperative angiography. **(D)** Follow-up angiography at 6 months showed aneurysm occlusion completely.

Treatment details, complications, and angiographic outcomes are summarized in Table 2. In the series of cases, only one patient experienced balloon angioplasty in the flow diverter group. More branches covered with stents were found in the conventional stent group (88.9 vs 33.3%, *p* = 0.008). Although the number of stents placed was significantly higher in the conventional stent group (1.57 ± 0.76 vs. 1.09 ± 0.29; *p* = 0.016), procedure time was similar in each group. The proportion of patients with OKM Grade A2 or A3 within the aneurysm immediately after stent placement was higher in the flow diverter stent group (95.5 vs. 57.1%; *p* = 0.004). Mean follow-up was similar in both groups. The proportion of patients with OKM Grade C or D on follow-up angiography was significantly higher in the flow diverter stent group (86.3 vs 50.0%; *p* = 0.047). Figures 1, 2 show representative VAFA cases treated using conventional and flow diverter stents, respectively. However, the incidence of parent artery stenosis or occlusion was also significantly higher in the flow diverter stent group (27.3% vs. 0; *p* = 0.032). The stenosis was mild in five of the six patients (83.3%) who developed stenosis. A case with a flow diverter (Figure 3) showed complete aneurysm obliteration, accompanied by parent artery stenosis at follow-up angiography. In addition, we further analyzed the patients with multiple stent technique, the technique was used in eight patients (two patients in the flow diverter group and six in the conventional stent group). The treatment and angiographic outcomes are detailed in Supplementary Table 2. A representative case with multiple stent technique is shown in Figure 4.

**Table 2:** Treatment details, complications, and angiographic and clinical outcomes.

	**Flow diverter stent (*n =* 22)**	**Conventional stent (*n =* 14)**	***P* value**
Branch covered with stent	5/15 (33.3)	8/9 (88.9)	0.008^*^
Number of stents	1.09 ± 0.29	1.57 ± 0.76	0.016^*^
Procedure time, min	94.46 ± 41.78	99.79 ± 34.59	0.592
Immediate angiographic results			0.004^*^
OKM Grade A1	1 (4.5)	6 (42.9)	
OKM Grade A2	10 (45.5)	7 (50.0)	
OKM Grade A3	11 (50)	1 (7.1)	
Complications, %	5 (22.7)	1 (7.1)	0.221
mRS at discharge	0.50 ± 0.67	0.21 ± 0.43	0.195
Mean follow-up (months)	7.77 ± 3.10	7.78 ± 8.31	0.144
Follow-up angiographic results			0.036^*^
OKM Grade A	1 (4.6)	6 (42.9)	
OKM Grade B	2 (9.1)	1 (7.1)	
OKM Grade C	1 (4.5)	1 (7.1)	
OKM Grade D	18 (81.8)	6 (42.9)	
Parent artery			0.032^*^
Patency, %	16 (72.7)	14 (100.0)	
Stenosis or occlusion, %	6 (27.3)	0 (0.0)	
Branches covered by stent			
Patency at follow-up	15/15 (100.0)	9/9 (100.0)	1.000
Follow-up mRS	0.09 ± 0.29	0.00 ± 0.00	0.252

* *P* < 0.05; FD, flow diverter; mRS, modified Rankin scale.

### Complications and clinical outcomes

Although the incidence of complications was higher in the flow diverter stent group (22.7 vs. 7.1%), the difference was not significant (*p* = 0.221). In patients with flow diversion, five patients developed thromboembolic complications during the procedure and post-procedure. In the conventional stent group, only one patient with thromboembolic complication was observed. The meanmRS score at discharge was higher in the flow diverter stent group, but the difference was not significant (0.50 ± 0.67 vs. 0.21 ± 0.43; *p* = 0.195). At the last clinical follow-up, the mean mRS score remained slightly higher in the flow diverter stent group but the difference was not significant (0.09 ± 0.29 vs. 0.00 ± 0.00; *p* = 0.252). Detailed complications and clinical outcome data are summarized in Table 2.

## Discussion

This study compared the safety and efficacy of flow diverters and conventional stents in patients with VAFAs who underwent SAT. In the flow diverter stent group, the rate of branches coming from the aneurysm was lower, and fewer stents were placed. Furthermore, the proportion of patients with significant or moderate stasis of flow on angiography performed immediately after stent placement and the rate of complete aneurysm obliteration on follow-up angiography was higher. However, the incidence of parent artery stenosis or occlusion was higher. Nonetheless, clinical outcomes and complication rates were similar between the flow diverter and conventional stent groups. These findings suggest that flow diverters might be beneficial for improving complete occlusion rate, while with an increased risk of mild parent artery stenosis.

### Current therapeutic strategies for VAFAs

Current surgical treatments for VAFAs include aneurysm trapping or wrapping and proximal ligation of the vertebral artery (10). However, they are not appropriate for all patients, depending on aneurysm location and size and brainstem blood flow considerations. High-flow bypass is another technically challenging option (11). Endovascular techniques for VAFAs can be divided into two approaches: deconstructive (trapping or proximal occlusion) and reconstructive (stent placement with or without coiling) (12). Deconstructive approaches are standard for hemorrhagic VAFAs because of their protection from rebleeding. Reconstructive approaches that preserve the parent artery are reserved for unruptured VAFAs with a low risk of rupture and patients whose contralateral vertebral artery is absent or hypoplastic. Sönmez et al. (12) performed a systematic review, suggested that reconstructive approaches may be as effective as possible and safer than deconstructive ones. Other studies have also reported that reconstructive approaches are feasible, safe, and effective (13–15). Endovascular reconstructive approaches to VAFAs include stent-assisted coil embolization and SAT using flow diverter or covered stents. Unlike saccular aneurysms, fusiform aneurysms have no aneurysm neck to stabilize coils; therefore, coiling requires stent assistance. Moreover, the mass effect from a coiled VAFA can be a problem, especially in large and giant ones (16). To remove this risk, SAT can be considered in selected cases (2, 6, 7, 15, 17).

### SAT of VAFAs

Before the introduction of flow diverter stents, conventional stents could only achieve a low degree of intra-aneurysmal flow reduction and were therefore unable to achieve complete thrombosis of fusiform aneurysms. The use of multiple stents within other stents was able to overcome this problem (17, 18), as the increased metal coverage of overlapping stents could achieve excellent flow diversion from the aneurysmal lumen. The difficulties in the deployment of overlapping stents would be important and need to be discussed. The difficulties consisted mainly of two points (technical difficulties with navigation through the first stent and delivering on the desired position). In our experience, a stent with a closed-loop design could be preferable, for which it is easily passed through curved blood vessels and the first stent. Moreover, regarding the landing location of the second stent, we attempted to deploy the second stent to make the overlap part of the two stents cover the aneurysm neck. The purpose of this was to increase the metal coverage rate of the aneurysm neck and produced a greater flow remodeling effect, that could reduce the possibility of aneurysm residual and recurrence in future. In addition, different from saccular aneurysms, the primary lesion of fusiform dissecting aneurysms was the proximal vascular injury of the aneurysm. Treatment should include the site of dissection and not only the aneurysmal dilatation. Thus, the stent needs to be sufficiently long to cover the entire vascular lesion. Since the development of flow diverter stents, they have been used to redirect flow in fusiform aneurysms to achieve high occlusion rates with low rates of recurrence. However, a potential complication of flow diverter use in the posterior circulation is devastating brainstem stroke from occlusion or stenosis of a critical arterial branch (19, 20). Our study directly compared complications and clinical and angiographic outcomes between flow diverter and conventional stents in patients with VAFAs who underwent SAT. Flow diverter stent placement achieved a higher complete occlusion rate and was associated with a higher rate of parent artery stenosis or occlusion; however, most cases of stenosis were mild. The placement of covered stents is also considered safe, feasible, and effective to treat VAFAs that are not associated with any critical side branches (15). However, their placement is often limited by their inflexibility and potential occlusion of side branches and perforators arising from the covered arterial segment.

### Theoretical mechanism of SAT

Stents can change or disrupt blood flow in a fusiform aneurysm, increase flow stagnancy, reduce inflow momentum, and decrease the impact zone under high flow (7, 21, 22). These phenomena are mainly caused by the following potential mechanisms. First, flow modifications arising from stent placement may have major hemodynamic effects that redirect flow along the normal course of the parent vessel, which disrupts flow into the aneurysm and promotes thrombosis. Second, the configuration of the parent vessel might change after stent placement, which may change the aneurysm inflow zone. Third, stent placement provides a scaffolding that supports neointimal overgrowth and promotes aneurysm thrombosis. Two previous studies have independently reported that stent placement modifies hemodynamic patterns in VAFAs such that thrombosis is favored and that the flow diversion effect of stenting is more significant when multiple overlapping stents are used (5, 23). Another study (21) reported that flow-diverter stents cause a better flow-diverting effect than conventional stents and result in greater reductions in flow, wall shear stress, and flow velocity. Lower flow velocity is indicative of stagnant blood flow, which can promote thrombosis and aneurysm occlusion. In our study, the rate of complete occlusion was higher in VAFAs treated with flow diverter stents than with conventional stents. Better flow diversion with the use of flow diverter stents might play a critical role in the treatment of fusiform aneurysms.

### Complications and clinical outcomes

In general, complications associated with endovascular aneurysm treatment are classified as hemorrhagic or thromboembolic. Ischemic complications, including perforator and downstream infarction, may occur in the acute and subacute stages. In a study of multiple stent therapy in patients with VAFAs, Chung et al. (17) reported a 100% success rate without treatment-related complications; in their literature review, they found a 3.6% complication rate in patients treated using multiple stents. Catapano et al. (24) also reported success and no complications with the use of flow diverter stents in patients with vertebral artery dissecting aneurysms. In contrast, Kallmes et al. (25) reported a 7.3% incidence of ischemic stroke in patients with posterior circulation aneurysms who underwent placement of a flow diverter stent (Pipeline embolization device). Perforator infarction comprises nearly half of the ischemic complications of flow diverter treatment and is usually explained by coverage of the perforator orifice by the stent or migration of disintegrated thrombus formed within the stent (26). Aneurysm location should be taken into consideration. For non-saccular aneurysms, the incidence of good neurologic outcome is higher with vertebral artery location than other locations; in contrast, mid/distal basilar artery aneurysms are associated with a higher risk of ischemic complications, probably because of the high density of perforators in this area (27). Our study only included patients with VAFAs and all branches covered by a stent were patent on follow-up angiography. Although six patients experienced a thromboembolic complication, all patients had a good or excellent clinical outcome. Moreover, the outcome did not significantly differ between groups. In a previous study, the incidence of in-stent stenosis was 38% in patients treated with the Silk Flow Diverter (Balt, Montmorency, France) and 39% in patients treated with the Pipeline embolization device (28). In-stent stenosis occurs because of a reaction of the arterial wall to the flow diverter device and usually can be detected on angiography within the first 2 months of placement. One previous study of flow diverter stent placement in vertebral artery dissecting aneurysms reported a 16.6% incidence of in-stent stenosis (29). Most cases of in-stent stenosis are mild to moderate and can be treated medically. The mechanism of in-stent stenosis following stent placement is probably multifactorial. As reported in previous studies, the underlying mechanism is likely related to stent malposition, intimal hyperplasia, antiplatelet agents, and adverse hemodynamics. Aguilar et al. explored the incidence, severity, and clinical course of in-stent stenosis after flow diverter stent, and they delineate that the potential role of malposition was associated with the occurrence of in-stent stenosis (30). Similarly, Ravindran et al. believe that stent malposition has been recognized as an important factor of in-stent stenosis and vascular injury, and inconsistent compliance between parent arteries and stents might cause intimal hyperplasia (31). Cohen et al. found that the occurrence of in-stent stenosis followed by improvement in the patients where the dose of antiplatelet agents was increased, and they believe that there is a unique biological behavior that is different from the in-stent stenosis associated with neointimal hyperplasia (28). The underlying molecular mechanism might be associated with the vascular smooth muscle cell stimulation due to intimal damage by the stent, which leads to the opening of stretch-responsive Ca2+ channels (31). In addition, there is evidence that hemodynamic factors also play a role in the progression of in-stent stenosis. Xiang et al. indicated that in-stent stenosis may result from torpid aneurysmal flow and exceptionally low wall shear pressure *via* unknown inflammatory pathways (32). In our study, we compared the occurrence of in-stent stenosis between flow diverter and conventional stents in patients with VAFAs after SAT and found a higher in-stent stenosis rate in patients with a flow diverter. However, the specific mechanism of in-stent stenosis ought to be further explored. High-precision imaging approaches might prove useful for the assessment of in-stent stenosis. Intravascular optical coherence tomography with a high resolution has emerged as a new method for evaluating the vessel wall and might enable gaining insight into the mechanism of stenosis. Xu et al. and Matsuda et al. enable *in vivo* visualization of intracranial arteries with applications in assessing neointima development and stenosis, such results support that optical coherence tomography might be helpful in gaining insight into the mechanism of in-stent stenosis in future (33, 34).

### Limitations

Our study has several limitations. First, the sample size was relatively small and the follow-up period was short. Large-scale multicenter prospective studies with longer follow-ups are warranted to confirm our findings. Second, different types and a number of stents may introduce bias, and the collection of two groups' subjects was not undertaken during the same period. Third, several other factors, such as platelet function and selection of stent, could also influence the results.

## Conclusion

SAT was safe and effective in patients with VAFAs. Flow diverter stents might be beneficial for improving complete occlusion rate than conventional stents with an increased risk of mild parent artery stenosis.

## Data availability statement

The raw data supporting the conclusions of this article will be made available by the authors, without undue reservation.

## Ethics statement

The studies involving human participants were reviewed and approved by the Ethics Committee of Beijing Tiantan hospital. The patients/participants provided their written informed consent to participate in this study.

## Author contributions

WL performed the statistical analysis and the manuscript writing. WL, WZ, YanmW, and YaZ acquired the data. WL, YiZ, and YangW contributed to data analysis and interpretation. WL, YiZ, and XL contributed to the experimental design, manuscript revision, handled funding, and supervision.

## Funding

This work was supported by the Henan Province Medical Science and Technology Research and Joint Construction Project (Grant No.: LHGJ20220337), the China Postdoctoral Science Foundation (Grant No.: 2022M712893), and the National Natural Science Foundation of China (Grant Nos.: 82201435, 81220108007, 81801156, 81801158, 81471167, 81671139, and 81960330).

## Conflict of interest

The authors declare that the research was conducted in the absence of any commercial or financial relationships that could be construed as a potential conflict of interest.

## Publisher's note

All claims expressed in this article are solely those of the authors and do not necessarily represent those of their affiliated organizations, or those of the publisher, the editors and the reviewers. Any product that may be evaluated in this article, or claim that may be made by its manufacturer, is not guaranteed or endorsed by the publisher.
