# Supplementary material for: Stent-alone treatment of unruptured vertebral artery fusiform aneurysms: A comparison of flow diverter and conventional stents
Source: Front Neurol. 2022 Nov 2;13:1012382. doi: 10.3389/fneur.2022.1012382 (PMC9666492; doi:10.3389/fneur.2022.1012382)
Supplement: Supplementary file 1 [file Data_Sheet_1.pdf]

Supplementary Table 1 The inclusion and exclusion criteria of the study.

Inclusion criteria:<br>1.aged more than 18 years and less than 80 years;<br>2.diagnosis of vertebral artery fusiform aneurysms by angiography;<br>3.treated with stent alone treatment;
Exclusion criteria:<br>1.diagnoses of arteritis, fibromuscular dysplasia, iatrogenic aneurysms, pseudoaneurysms or vertebrobasilar dolichoectasia;<br>2.are underwent simultaneous treatment for other cerebrovascular diseases, such as intracranial arteriovenous malformation and intracranial arteriovenous fistula;<br>3.have already been treated using endovascular approach for the vertebral artery fusiform aneurysms previously;

Supplementary Table 2 The treatment and angiographic outcomes of the patients with multiple stent technique

Group	Patient No	Age	Sex	Treatment	Follow-up
Flow diverter	Case 1	57	male	2 FD	OKM Grade D
	Case 2	47	male	2 FD	OKM Grade D
Conventional stent	Case 3	50	male	3 Enterprise	OKM Grade C
	Case 4	52	Male	2 Enterprise + 1 Solitaire	OKM Grade B
	Case 5	50	male	1 Enterprise +1 Solitaire	OKM Grade B
	Case 6	60	male	2 Enterprise	OKM Grade B
	Case 7	51	male	2 LVIS	OKM Grade D
	Case 8	60	male	2 Enterprise	OKM Grade B
